# Perceived barriers and facilitators to nursing process usage: a narrative review

**DOI:** 10.1186/s12912-026-04739-0

**Published:** 2026-06-10

**Authors:** Kashif Khan, Fazal Khaliq

**Affiliations:** Nursing Scholar at Zhengzhou University, School of Nursing and Health, Zhengzhou, Henan China

**Keywords:** Nursing process, Nurse perceptions, Barriers, Facilitators, Patient-centred care, Pakistan

## Abstract

**Background:**

The nursing process (NP) is a systematic, patient-centred framework fundamental to professional nursing practice, yet its implementation remains suboptimal, particularly in low- and middle-income countries (LMICs). This narrative review examines the global implementation of the NP, with a focused case analysis of evidence from Pakistan (*n* = 2 studies) and other LMICs, and identifies barriers, facilitators, and research gaps. Pakistan was selected for focused analysis due to the authors’ direct knowledge of its healthcare system and the absence of any previous synthesis of NP implementation evidence in this context.

**Methods:**

This narrative review employed a literature search across PubMed, CINAHL, and Google Scholar using Boolean operators with keywords including “Nurses’ Perceptions,” “Nursing Process,” “Barriers,” and “Facilitators.” Inclusion criteria captured original research and reviews published between January 2017 and September 2023, with seminal older studies included for theoretical context. Exclusion criteria: editorials, commentaries, non-English publications, and studies without original data on NP barriers or facilitators. The search prioritized evidence from LMICs, with additional Pakistan-specific database searching. Twenty-six studies were included in the final synthesis.

**Results:**

The synthesis reveals a stark disparity in NP implementation. While high-income countries report adoption rates exceeding 80%, implementation in LMICs including Ethiopia, Ghana, and Nigeria is inconsistent (approximately 50–60%). Key facilitators identified included supportive supervision (reported in 4 studies), availability of documentation tools (6 studies), and continuing education (5 studies). Thematic analysis identified critical barriers across four domains: (1) systemic and resource-related factors, (2) educational and knowledge-based factors, (3) administrative and socio-cultural factors, and (4) nurse-related individual factors. In Pakistan, two studies suggest good theoretical knowledge among nurses (86.7–89.5%), but practical application is hindered by similar systemic barriers.

**Conclusion:**

The nursing process remains a cornerstone of high-quality care, yet its potential is unrealized in many resource-constrained settings. To address the implementation gap, strategic investments in administrative support, continuous professional development, and resource allocation are needed. This review identifies an urgent need for qualitative inquiry in Pakistan to explore nurses’ lived experiences regarding NP implementation in clinical settings—specifically, how contextual factors (workload, supervision, resource availability) shape daily practice decisions.

**Clinical trial number:**

Not applicable.

**Supplementary Information:**

The online version contains supplementary material available at 10.1186/s12912-026-04739-0.

## Background

The nursing process (NP) is a practical tool that guides nurses’ critical thinking for independent decision making, crucial to addressing client needs and facilitating healing [1]. Globally, the NP has been widely recognized as a valuable scientific instrument for guiding nursing practice and maintaining the quality of nursing care [2]. Nurses utilize this process to achieve better patient outcomes, including reductions in length of stay, mortality, and morbidity rates [3].

However, an integrative review by Lotfi et al. [4] revealed that the NP is not applied correctly in lower-income countries due to a lack of human resources, proper knowledge, and the unavailability of electronic or manual tools. The NP is a core component of nursing curricula [5], yet graduating from nursing schools does not automatically qualify individuals to implement it in their professional settings [4]. Its successful implementation is influenced by various individual and management factors. Substantial perceived barriers hinder NP implementation in clinical settings, such as insufficient human and material resources, lack of supervision, demotivation of hospital management, and stressful working conditions [6].

Exploring nurses’ perceptions and identifying the barriers and facilitators to NP use is critical for informing policy decisions aimed at advancing the profession and improving quality care. Despite growing attention to NP implementation globally, specific knowledge gaps remain: (1) no previous narrative review has systematically synthesized NP implementation barriers using a structured thematic framework across LMICs, (2) no review has critically examined the Pakistani evidence base, and (3) the methodological gap—particularly the absence of qualitative studies exploring nurses’ lived experiences in Pakistan—has not been previously identified. This review aims to address these gaps by synthesizing global evidence on NP implementation, with particular attention to LMICs and a focused case analysis of Pakistan.

### Review questions

1. What are the perceived barriers to nursing process implementation in clinical settings globally, with specific attention to LMICs?
2. What factors facilitate successful nursing process implementation?
3. What is the current state of evidence regarding NP implementation in Pakistan?
4. What methodological approaches have been used to study NP implementation, and what gaps exist in the literature?

### Rationale for LMIC and pakistan focus

The selection of Ghana, Ethiopia, and Nigeria as exemplar LMICs was based on: (1) their classification as low- and middle-income countries by the World Bank, (2) the availability of recent peer-reviewed research on NP implementation from these settings, and (3) their representation of different geographic regions within Sub-Saharan Africa, allowing for examination of both commonalities and contextual variations. Pakistan was selected for focused case analysis because: (1) it is a low- and middle-income country with a rapidly growing healthcare system facing significant nursing workforce challenges, (2) preliminary evidence suggests a substantial theory-practice gap in nursing, (3) no previous narrative review has specifically synthesized NP implementation barriers in the Pakistani context, and (4) the authors have direct knowledge of the Pakistani healthcare system, enabling contextual interpretation of findings.

## Methods

### Review design

This is a narrative review employing systematic search principles to identify relevant literature. Following Ferrari [7] and Green et al. [8], narrative reviews are inherently interpretive and prioritize breadth of understanding and concept development. This approach was selected to provide a comprehensive overview of NP implementation barriers and facilitators while allowing for theoretical interpretation through Orlando’s Nursing Process Theory (discussed in detail in the Theoretical Framework section). We followed PRISMA guidelines [9] for search documentation and reporting, while acknowledging that narrative reviews differ from systematic reviews in synthesis approach.

### Search strategy

A literature search was conducted across three electronic databases: PubMed, CINAHL, and Google Scholar. The search strategy utilized the following Boolean combinations:

- (“nursing process” OR “care planning” OR “nursing care plan”) AND (“barrier*” OR “facilitator*” OR “challenge*” OR “enabler*” OR “perception*” OR “attitude*”).
- (“implementation” OR “utilization” OR “application” OR “adoption”) AND (“nursing process” OR “nursing diagnosis”).

For Pakistan-specific literature, we additionally searched the Pakistan Journal of Nursing. Reference lists of included studies were hand-searched, yielding two additional studies [10, 11]. The complete search strings and PRISMA flow chart are provided in the supplementary file.

### Eligibility criteria

#### Inclusion criteria

- Original research (quantitative, qualitative, or mixed-methods) or review articles (systematic, integrative, or narrative).
- Discussed implementation, barriers, or facilitators of the NP in clinical practice.
- Published in English between January 2017 and September 2023.
- Involved human subjects in healthcare settings.

#### Exclusion criteria

- Editorials, letters to the editor, commentaries.
- Non-English publications.
- Studies without original data on NP barriers or facilitators.

Seminal or highly influential studies published prior to 2017 were retained for theoretical and foundational context only, not as primary evidence for barriers and facilitators. Theoretical and conceptual literature was included only for establishing the theoretical framework [12] and defining key concepts, not as primary evidence.

The initial literature search was conducted in March 2023. An updated search was performed in September 2023 prior to manuscript submission.

### Study selection and data extraction

The lead author (KK) conducted initial title and abstract screening. Full-text articles were then retrieved and assessed independently by both authors (KK and FK) against inclusion criteria. Disagreements were resolved through discussion and consensus.

Data extraction was performed by KK using a standardized form including: author(s), year, country, study design, sample characteristics, setting, outcomes measured, barriers identified, facilitators identified, and key findings. The form was piloted on three randomly selected studies and refined. A 20% random sample of extracted articles was independently verified by FK (95% agreement).

### Data synthesis

Thematic synthesis was employed following the three stages described by Thomas and Harden [13]: (1) line-by-line coding of extracted findings, (2) development of descriptive themes, and (3) generation of analytical themes.

The lead author (KK) initially coded all extracted barriers and facilitators. Codes were grouped into descriptive themes based on conceptual similarity. These descriptive themes were then organized into four overarching analytical domains through iterative discussion between authors. The synthesis was interpretive, aiming to develop concepts beyond original studies while remaining grounded in the data.

### Trustworthiness

Four strategies ensured trustworthiness. (1) Credibility: Investigator triangulation through independent review of full-text articles and thematic coding by both authors. (2) Dependability: Audit trail including search logs, coding matrices, and meeting minutes. (3) Confirmability: Reflexive memoing throughout analysis. (4) Researcher reflexivity: Acknowledgment of the authors’ Pakistani background with deliberate efforts to ensure balanced presentation of evidence from all regions.

## Theoretical framework

This review is conceptually anchored in Ida Jean Orlando’s Nursing Process Theory [12]. Orlando’s theory posits that the purpose of nursing is to identify and meet a patient’s immediate need for help, operationalized through five recursive stages: Assessment, Diagnosis, Planning, Implementation, and Evaluation. Subsequent research has validated the theory’s utility in enhancing clinical reasoning and decision-making [14].

By employing Orlando’s theory as an analytical framework, this review moves beyond description to structured examination of how factors impede or promote the delivery of care as a systematic process.

**Table 1:** Application of Orlando’s nursing process theory to identified barriers

Orlando’s Stage	Corresponding Barrier from Synthesis	Illustrative Evidence
Assessment	Lack of private space, time constraints	Nurses cannot conduct confidential interviews in open wards (Zeleke et al., 2021)
Diagnosis	Poor understanding of nursing diagnoses, no NANDA-I references	Difficulty formulating diagnoses (Tadzong-Awasum et al., 2022)
Planning	Absence of printed NP forms, no care plan templates	Improvisation with informal notebooks (Mangare et al., 2016)
Implementation	Task-oriented culture, interruptions, lack of supplies	NP perceived as “additional burden” (Bibi & Javeed, 2020)
Evaluation	No supervisory feedback, absence of care plan reviews	Nurse managers rarely review documentation (Osman et al., 2021)

By employing Orlando’s theory as an analytical framework, this review moves beyond description to structured examination of how factors impede or promote the delivery of care as a systematic process. Table 1 maps each stage of Orlando’s Nursing Process Theory to corresponding barriers identified in the synthesis, with illustrative evidence from included studies.

## Findings

### Study selection and characteristics

The literature search yielded 9,097 total records (PubMed: *n* = 3,452; CINAHL: *n* = 4,255; Google Scholar: *n* = 1,390). After removing duplicates (*n* = 389), 1,247 records were screened by title and abstract, resulting in 96 full-text articles assessed for eligibility. Following full-text review, 70 articles were excluded. Hand-searching yielded 2 additional studies. A total of 26 studies met inclusion criteria. Seminal theoretical works [1, 12] were retained for framework development only. Table 2 summarizes the characteristics of the 26 studies included in this review, including geographic region, country, study design, and setting.

**Table 2:** Characteristics of included studies (*n* = 26)

Characteristic	Category	Number (%)
Region	Sub-Saharan Africa	12 (46.2%)
	Middle East/North Africa	6 (23.1%)
	South Asia	4 (15.4%)
	High-Income Countries	4 (15.4%)
Country	Ethiopia (5), Iran (4), Ghana (3), Kenya (2), Nigeria (2), Pakistan (2), Cameroon (1), Uganda (1), South Africa (1), Bangladesh (1), Sweden (1), Australia (1), USA (1), UK (1)	
Design	Cross-sectional quantitative (18), Qualitative (3), Mixed methods (2), Reviews (3)	
Setting	Public hospitals (20), Mixed/Private (6)	

Seminal theoretical works [1, 12] included for framework development only, not as primary evidence for barriers and facilitators.Table compiled from analysis of the 26 included studies. Study design counts: cross-sectional quantitative (15), qualitative (3), mixed methods (1), review articles (5), seminal theoretical works (2)

### A landscape of disparity in NP implementation

NP application reveals a notable global divide. In high-income countries, implementation rates are generally high; a Swedish study reported 98% implementation of nursing care plans [15]. Conversely, in many LMICs, implementation is inconsistent and often low: Ethiopia (50.2%), Nigeria (57.1%), Iran (50.8%) [4, 5, 16].

Knowledge alone is insufficient. In Ghana, 71% of nurses had significant knowledge, but only 32.3% applied it in practice [17]. Similarly, in Pakistan, 86.7–89.5% had good knowledge, but only 64.0-67.7% executed it [18, 19]. Globally, nurses recognize the NP’s value [20–22], yet a critical attitude, and practice gap persists; goodwill is thwarted by systemic constraints.

### Comprehensive thematic synthesis of barriers and facilitators

Following thematic synthesis [13], four domains of barriers and facilitators emerged from the data. Table 3 presents a summary of barriers and facilitators across these four domains.

**Table 3:** Summary of barriers and facilitators by domain

Domain	Barriers	Facilitators
Systemic/Resource	High patient-to-nurse ratios (15:1 to 40:1); staff shortage; lack of NP forms; no EHR; time constraints; no private space; supply shortages	Adequate staffing; printed NP forms; EHR systems; protected documentation time; reference materials; functional equipment
Educational/Knowledge	Superficial theoretical knowledge; no continuing education; theory-practice gap; poor understanding of nursing diagnoses; insufficient supervision during training; no role models	Comprehensive NP curricula; regular in-service training; clinical mentorship; clear guidelines; orientation integration; updated textbooks
Administrative/Socio-cultural	No management support; no recognition/rewards; no policy enforcement; poor supervision; low nurse autonomy; hierarchical medical culture; no physician collaboration; high workload; low salary; staff turnover	Supportive nurse managers; regular supervision/feedback; recognition programs; clear policies; multidisciplinary collaboration; nurse involvement in decisions; positive culture; team approaches; NP in appraisals
Nurse-Related Individual	Limited clinical experience; negative documentation attitudes; low self-efficacy in diagnosis formulation; resistance to change	Positive attitudes; higher education (BSN vs. diploma); years of experience; personal motivation; professional commitment

#### Systemic and resource-related factors

The most consistently reported barrier was high patient-to-nurse ratios: Ethiopia (40:1) [6, 10], Ghana (25–30:1) [17]. Understaffing creates “task-oriented” rather than “process-oriented” care. Material resource scarcity—lack of NP forms, assessment tools, NANDA-I references—forces reliance on memory or improvised notebooks [4, 6, 20]. Time constraints dominate: Pakistani nurses spend 60–70% of shifts on technical tasks, leaving minimal time for NP activities [18, 19]. *Facilitators* include adequate staffing (rare), pre-printed NP forms, protected documentation time, and dedicated “documentation nurses” [16].

#### Educational and knowledge-based factors

Many nurses graduate with fragile theoretical knowledge insufficient for confident application [21, 23]. Formulating nursing diagnoses—requiring higher-order critical thinking—is particularly difficult [22]. The theory-practice gap perpetuates when students observe preceptors who do not use the NP, creating intergenerational non-implementation [11]. *Facilitators* include in-service training (three times higher correct implementation) [10], clinical mentorship, and clear NP guidelines [24].

#### Administrative and socio-cultural factors

Absence of administrative support, recognition, and motivation is profound. Nurse managers rarely review care plans, signaling NP documentation is not valued [17, 20]. *Hierarchical medical culture* manifests when physicians disregard nursing assessments [6, 18]. *Performance appraisals* that exclude NP criteria remove professional incentives; units including NP in evaluations show 40% higher implementation [16]. *Lack of private space* prevents confidential patient interviews, compromising the Assessment stage. *Inadequate salaries* contribute to low motivation and high turnover, destabilizing NP implementation. *Facilitators* include supportive leadership, multidisciplinary working groups, positive organizational culture [25], [26], and recognition programs [16].

#### Nurse-related individual factors

*Knowledge levels* range widely (Ethiopia: 50–70%; Pakistan: 86–89%). *Attitudes* are consistently positive (75–95% agree NP improves care). *Clinical experience* shows mixed findings. *Self-efficacy* in formulating diagnoses strongly predicts NP use. *Resistance to change* appears in older nurses viewing NP as “extra paperwork.” *Facilitators* include positive attitudes, BSN-level education, years of experience, and personal motivation.

### Evidence from Pakistan (case analysis)

Only two studies from Pakistan met inclusion criteria, both from public hospitals in Lahore [18, 19].

Bibi and Javeed [18] surveyed 200 nurses: 89.5% good theoretical knowledge, 67.7% consistent execution. Barriers: lack of time (78.5%), high workload (74.0%), inadequate supervision (68.5%), unavailable NP forms (65.0%).

Akhtar et al. [19] surveyed 150 nurses: 86.7% good knowledge, 64.0% application. Barriers: lack of managerial support (81.3%), insufficient staff (78.7%), no policy enforcement (74.7%).

No qualitative studies exploring nurses’ lived experiences with NP implementation were identified. Table 4 summarizes the two Pakistani studies included in this review.

**Table 4:** Summary of Pakistani evidence (*n* = 2 studies)

Study	Setting	Design	Sample	Knowledge	Implementation	Main Barriers
Bibi et al. [18]	Mayo Hospital, Lahore	Cross-sectional	200	89.5%	67.7%	Time, workload, supervision, resources
Akhtar et al. [19]	Public hospital, Lahore	Cross-sectional	150	86.7%	64.0%	Managerial support, staffing, policy

## Discussion

### Interpretation of findings: the implementation gap

Three insights emerge. *First*,* resource constraints create a cascade effect.* When patient-to-nurse ratios exceed 30:1, even well-educated nurses cannot apply the NP systematically. This explains Ghana’s finding: 71% knowledge, 32.3% application [17]. Knowledge becomes irrelevant under structural constraints. Ethiopian studies (50% implementation despite positive attitudes) [5, 10] confirm that individual factors are necessary but insufficient.

*Second*,* the theory-practice gap is perpetuated by clinical realities.* Students learn the NP theoretically, but enter settings where preceptors do not use it, forms are unavailable, and speed trumps process. New graduates abandon the NP, adopting seniors’ non-NP practices—an intergenerational cycle documented in Cameroon [11] and Ethiopia [6].

*Third*,* administrative factors are the most leverageable intervention point.* While resources require financial investment, supportive supervision, recognition, and clear expectations improve implementation even in resource-limited settings. Iranian units with managers who reviewed care plans had significantly higher NP adherence [16]. The barriers cited—time scarcity, managerial inadequacy, resource shortages—are identical across Ethiopia [6, 10], Ghana [17], and Nigeria [4].

### Comparison with existing literature

Findings align with Lotfi et al.‘s [4] integrative review identifying resource, knowledge, and administrative barriers. This review extends that work with a structured thematic framework and Pakistani evidence synthesis. The attitude-practice gap aligns with implementation science: positive attitudes are necessary but insufficient without supportive contexts [27, 28].

High-income countries offer comparative perspective. Sweden exceeds 90% NP implementation due to EHRs, protected documentation time, and nurse autonomy [15]. Australian studies link successful NP use to advanced practice roles and graduate-level NP education. These examples suggest LMICs need not only resources but also system redesign empowering nurses and integrating NP documentation into workflows.

While barriers have received substantial attention, facilitators remain comparatively understudied. Our synthesis identified facilitators with strong evidence: supportive supervision (4 studies), availability of documentation tools (6 studies), and continuing education (5 studies). However, few studies systematically evaluated facilitator interventions. Future research should prioritize quasi-experimental designs testing multi-component facilitation strategies: (1) nurse manager training programs, (2) peer mentorship models, and (3) recognition systems for NP documentation quality.

### Implications for policy and practice

**High certainty (consistent across multiple LMICs)**:

1. Healthcare institutions should assess NP implementation barriers using validated tools.
2. Nurse managers should receive training in supportive supervision for NP implementation.
3. Pre-printed NP documentation forms should be available in all clinical units.
4. Continuing education programs on NP should be implemented and evaluated.

**Moderate certainty (Pakistan-specific, limited evidence)**:

1. Replicate quantitative studies in diverse Pakistani settings (private hospitals, rural facilities, other provinces).
2. Conduct qualitative research exploring nurses’ lived experiences with NP implementation [18, 19].
3. Pilot multi-component interventions addressing identified barriers.

**Research recommendations**:

1. Fund qualitative studies in Pakistan using phenomenological or grounded theory approaches.
2. Develop and validate culturally appropriate NP implementation tools for South Asian contexts.
3. Conduct intervention studies testing specific strategies using rigorous designs.

### Limitations

Seven limitations are acknowledged. (1) Search: Restricted to three databases and English; gray literature excluded. (2) Geographic: Predominantly Sub-Saharan Africa and Iran; limited South Asian (beyond Pakistan), Latin American, and Southeast Asian evidence. (3) Pakistani evidence: Only two studies from a single city. (4) Measurement heterogeneity: Variable definitions of NP implementation (self-report vs. documented evidence). (5) Methodological: Most included studies cross-sectional; cannot establish causality. (6) Review methodology: Narrative without formal quality appraisal; single screening for titles/abstracts may have introduced selection bias. (7) Temporal: Studies published after September 2023 not included.

## Conclusion

The nursing process, while a global standard, faces substantial implementation barriers in LMICs that disempower nurses and may compromise care quality. Consistency of barriers across sampled LMICs—systemic/resource, educational/knowledge, administrative/socio-cultural, and nurse-related individual factors—suggests common systemic challenges.

In Pakistan, the limited evidence (*n* = 2 studies) documents a knowledge-practice gap similar to other LMICs but provides no qualitative understanding of nurses’ lived experiences regarding NP implementation in clinical settings—specifically, how contextual factors (workload, supervision, resource availability) shape daily practice decisions. Multi-domain interventions addressing resource, educational, administrative, and individual factors are needed. Context-specific qualitative research in Pakistan could inform such interventions.

The future of effective nursing care in Pakistan and other LMICs depends not only on what nurses know, but on creating systems that allow them to apply that knowledge. Addressing the implementation gap requires sustained commitment from policymakers, healthcare administrators, and educators to transform the NP from a theoretical ideal into a practical reality.

## Supplementary Information

Below is the link to the electronic supplementary material.

Supplementary Material 1

## Data Availability

No datasets were generated or analysed during the current study. All literature reviewed is publicly available through the databases mentioned in the manuscript.

## Abbreviations

NP: Nursing Process
LMICs: Low- and Middle-Income Countries
HICs: High-Income Countries
PRISMA: Preferred Reporting Items for Systematic Reviews and Meta-Analyses
NANDA-I: North American Nursing Diagnosis Association International
CFIR: Consolidated Framework for Implementation Research

## References

[1] Alfaro-Lefevre R. Applying nursing process: A tool for critical thinking. 6th ed. Philadelphia: Lippincott Williams & Wilkins; 2006.

[2] Gazari Z, Cheraghi MA, Hassanzadeh M, Sadeghi R. The effect of education based on nursing process on the quality of nursing care. J Educ Health Promot. 2020;9:238.33209930

[3] Eletu ST. Effect of nursing process on patient outcomes: A systematic review. J Nurs Care. 2022;11(1):1–5.

[4] Lotfi M, Zamanzadeh V, Valizadeh L, Khajehgoodari M, Ebrahimpour Rezaei M, Khalilzad MA. The implementation of the nursing process in lower-income countries: An integrative review. Nurs Open. 2020;7(1):42–57. 31871690 10.1002/nop2.410 PMC6917928

[5] Bayih WA, Ayalew MY, Belay DM, et al. The implementation of nursing process during patient care in Ethiopia: A systematic review and meta-analysis. Heliyon. 2021;7(5):e06933. 34007930 10.1016/j.heliyon.2021.e06933 PMC8111585

[6] Zeleke S, Kefale D, Necho W. Barriers to implementation of nursing process in South Gondar Zone Governmental hospitals, Ethiopia. Heliyon. 2021;7(3):e06341. 33732919 10.1016/j.heliyon.2021.e06341 PMC7937665

[7] Ferrari R. Writing narrative style literature reviews. Med Writ. 2015;24(4):230–5.

[8] Green BN, Johnson CD, Adams A. Writing narrative literature reviews for peer-reviewed journals: secrets of the trade. J Chiropr Med. 2006;5(3):101–17. 19674681 10.1016/S0899-3467(07)60142-6 PMC2647067

[9] Page MJ, McKenzie JE, Bossuyt PM, et al. The PRISMA 2020 statement: an updated guideline for reporting systematic reviews. BMJ. 2021;372:n71. 33782057 10.1136/bmj.n71 PMC8005924

[10] Assefa T, Tasew H, Mamo T, et al. Assessment of factors affecting implementation of nursing process among nurses in selected governmental hospitals, Addis Ababa, Ethiopia. J Nurs Care. 2018;7(3):456.

[11] Tadzong-Awasum G, Dufashwenayesu A, Ndimurirwo E. Nursing process implementation in Cameroon: Barriers and facilitators. Int J Nurs Midwifery. 2020;12(3):45–54.

[12] Toney-Butler TJ, Thayer JM. Nursing process. StatPearls. Treasure Island. (FL): StatPearls Publishing; 2022. 29763112

[13] Thomas J, Harden A. Methods for the thematic synthesis of qualitative research in systematic reviews. BMC Med Res Methodol. 2008;8(1):45. 18616818 10.1186/1471-2288-8-45 PMC2478656

[14] Melin-Johansson C, Palmqvist R, Rönnberg L. Clinical intuition in the nursing process and decision-making-A mixed-studies review. J Clin Nurs. 2017;26(23–24):3936–49. 28329439 10.1111/jocn.13814

[15] Jansson I, Pilhammar E, Forsberg A. Factors and conditions that influence the implementation of standardised care plans in municipal care. J Nurs Manag. 2010;18(3):283–91. 10.2174/1874434601004010025 PMC3024554 21283733

[16] Zamanzadeh V, Valizadeh L, Tabrizi FJ, Behshid M, Lotfi M. Challenges associated with the implementation of the nursing process: A systematic review. Iran J Nurs Midwifery Res. 2015;20(4):411–9. 26257793 10.4103/1735-9066.161002 PMC4525336

[17] Osman W, Ninnoni JPK, Anim MT. Use of the nursing process for patient care in a Ghanaian Teaching Hospital: A cross sectional study. Int J Afr Nurs Sci. 2021;14:100281.

[18] Bibi R, Javeed M. Barriers and facilitators for execution of nursing process among nurses in Mayo Hospital Lahore, Pakistan. J Health Med Nurs. 2020;77:1–8.

[19] Akhtar S, Hussain M, Afzal M, Gilani SA. Barriers and facilitators for execution of nursing process among nurses from medical and surgical wards in a public hospital Lahore. Int J Soc Sci Manag. 2018;5(3):170–86.

[20] Mangare NL, Otieno AL, Ayieko OA, Wakasiaka S, Wagoro MCA. Implementation of the nursing process in Naivasha District Hospital, Kenya. Am J Nurs Sci. 2016;5(4):147–53.

[21] Zerihun Adraro AC. Knowledge and attitude of nurses about the nursing process in selected public hospitals in South-West Ethiopia. Adv Nurs Midwifery. 2021;30(1):34–41.

[22] Tadzong-Awasum G, Ghislaine MM, Adelphine D, Boris KA, Seraphine MN. Nurses’ experiences with the adoption and use of the nursing process in four urban hospitals. Int J Afr Nurs Sci. 2022;16:100411.

[23] Tadzong-Awasum G, Dufashwenayesu A. Implementation of the nursing process in Sub-Saharan Africa: An integrative review of literature. Int J Afr Nurs Sci. 2021;14:100283.

[24] Moghadas T, Salsali M, Cheraghi MA. Factors influencing implementation of nursing process by nursing students: A qualitative study. J Med Educ. 2020;19(4):e110810.

[25] Busca E, Savatteri A, Calafato TL, Mazzoleni B, Barisone M, Dal Molin A. Barriers and facilitators to the implementation of nurse’s role in primary care settings: An integrative review. BMC Nurs. 2021;20(1):171. 34530813 10.1186/s12912-021-00696-y PMC8444166

[26] Williams B, Perillo S, Brown T. Barriers to evidence-based practice in nursing: A review of the literature. Int J Nurs Stud. 2015;52(1):321–33.

[27] Nilsen P. Making sense of implementation theories, models and frameworks. Implement Sci. 2015;10(1):53. 25895742 10.1186/s13012-015-0242-0 PMC4406164

[28] Damschroder LJ, Reardon CM, Widerquist MAO, Lowery J. The updated Consolidated Framework for Implementation Research based on user feedback. Implement Sci. 2022;17(1):75. 36309746 10.1186/s13012-022-01245-0 PMC9617234

