# Supplementary material for: Perceived barriers and facilitators to nursing process usage: a narrative review
Source: BMC Nurs. 2026 Jun 10;25:724. doi: 10.1186/s12912-026-04739-0 (PMC13480092; doi:10.1186/s12912-026-04739-0)
Supplement: Supplementary file 1 [file 12912_2026_4739_MOESM1_ESM.docx]

Figure 1. PRISMA Flow Chart of Literature Search Strategy

Duplicates Removed

(n = 389)

PubMed	CINAHL	Google Scholar

(n = 3,452)	(n = 4,255)	(n = 1,390)

Total Records Identified	(n = 9,097)

Indentation

Eligibility

Records Excluded	(n = 1,151)

Records Screened by Title/Abstract (n = 1,247)

Analysis

Full-Text Articles Assessed	for Eligibility	(n = 96)

Not original research or review (n=24)

No NP barriers/facilitators (n=31)

Non-English(n=8)

Duplicate publications (n=7)

Additional Records from Hand-Searching (n = 2)

Full-Text Articles Excluded (n = 70)

Number of articles finally reviewed=26

Quantitative=15

Qualitative= 3

Mixed Method= 1

Review Articles= 5

Books= 2*

*Books included for theoretical framework only, not primary evidence

Inclusion
